# Optimized polymer enhanced foam flooding for ordinary heavy oil reservoir after cross-linked polymer flooding

**DOI:** 10.1007/s13202-015-0226-2

**Published:** 2016-01-08

**Authors:** Chen Sun, Jian Hou, Guangming Pan, Zhizeng Xia

**Affiliations:** 1 State Key Laboratory of Heavy Oil Processing, China University of Petroleum, Qingdao, Shandong 266580 China; 2 College of Petroleum Engineering, China University of Petroleum, Qingdao, Shandong 266580 China; 3 College of Chemical Engineering, China University of Petroleum, Qingdao, Shandong 266580 China; 4 Bohai Petroleum Research Institution, Tianjin Branch of CNOOC Ltd., Tianjin, China

**Keywords:** Natural gas, Polymer enhanced foam flooding, Oil displacement experiment, Selective plugging

## Abstract

A successful cross-linked polymer flooding has been implemented in JD reservoir, an ordinary heavy oil reservoir with high permeability zones. For all that, there are still significant volumes of continuous oil remaining in place, which can not be easily extracted due to stronger vertical heterogeneity. Considering selective plugging feature, polymer enhanced foam (PEF) flooding was taken as following EOR technology for JD reservoir. For low cost and rich source, natural gas was used as foaming gas in our work. In the former work, the surfactant systems CEA/FSA1 was recommended as foam agent for natural gas foam flooding after series of compatibility studies. Foam performance evaluation experiments showed that foaming volume reached 110 mL, half-life time reached 40 min, and dimensionless filter coefficient reached 1.180 when CEA/FSA1 reacted with oil produced by JD reservoir. To compare the recovery efficiency by different EOR technologies, series of oil displacement experiments were carried out in a parallel core system which contained cores with relatively high and low permeability. EOR technologies concerned in our work include further cross-linked polymer (C-P) flooding, surfactant-polymer (S-P) flooding, and PEF flooding. Results showed that PEF flooding had the highest enhanced oil recovery of 19.2 % original oil in place (OOIP), followed by S-P flooding (9.6 % OOIP) and C-P flooding (6.1 % OOIP). Also, produced liquid percentage results indicated PEF flooding can efficiently promote the oil recovery in the lower permeability core by modifying the injection profile.

## Introduction

Currently, chemical flooding has been widely used in ordinary heavy oil reservoirs in China (Zhou et al. 2006, 2013; Gao 2011; Hou et al. 2013a, b; Zhang et al. 2014). As one of the main techniques, cross-linked polymer flooding has been widely implemented for enhancing oil recovery of high water cut reservoirs and achieved good development effect (Urbissinova et al. 2010; Renouf 2014). Yet for all that, there are still significant volumes of oil remaining in place. In most cases, however, 40–50 % of the original oil in place (OOIP) can not be easily extracted due to stronger reservoir heterogeneity and more complicated plane distribution characteristic. Along with further promotion and application of polymer flooding technology, similar remaining oil reserves will rise continually.

Many cases showed that it played a limited role in oil recovery improvement to continue using polymer as a profile control and flooding agent in different chemical methods after cross-linked polymer flooding, such as secondary cross-linked polymer flooding, surfactant-polymer (SP) flooding and alkali-surfactant-polymer (ASP) flooding. That is mainly due to the further development of thief channels. The number of thief channels increased and the distribution of remaining oil became more complex after the first cross-linked polymer flooding (Maghzi et al. 2014). Besides, stronger heterogeneity of post cross-linked polymer flooding reservoirs makes injected fluid prefer to flow through highly permeable thief channels, rather than displace the remaining oil in low permeability areas,where a mass of remaining oil locates as continuous state.

A successful cross-linked polymer flooding has been implemented in the target oil field (Yang et al. 2008), JD reservoir, an ordinary heavy oil reservoir with high permeability zones. After cross-linked polymer flooding, the distribution of remaining oil became more complicated and it was more difficult to use the chemical flooding which taking only polymer as a profile control for enhancing its oil recovery. Therefore, a new kind of profile control and flooding agent with greater sweep efficiency is of great importance for the development of post-polymer flooding reservoirs.

Foam flooding using foam generated by a mixture of gas (nitrogen, natural gas or other gases) and foaming agents as an oil displacement medium (Pang 2010; Chen et al. 2010; Li et al. 2010). A lot of works reported that enhanced foam flooding was widely used to further develop this kind of post-polymer flooding reservoirs (Li et al. 2009). Generally, nitrogen was used as foaming gas because of its good compatibility with foam agent (Zitha and Du 2010; Hou et al. 2013a, b). However, nitrogen supply needs to increase nitrogen production equipment, which is a big investment for the kind of high water cut reservoirs. There was rich natural gas produced by JD reservoir. Also for low cost and good economy, natural gas was used as foaming gas in our work. However, compared with nitrogen, it is more difficult to select foam agent for natural gas. In the former work (Pan et al. 2013), foam Agent CEA:FSA1(7:3) was selected for natural gas foam flooding. Considering the evaluation indexes of foaming capability, foam half-life time and interfacial tension, CEA:FSA1(7:3) and CEA:DHF-1(7:3) were chosen by laboratory experiments conducted under simulated water–oil conditions of JD Reservoir. And, further foam performance evaluation experiments showed that CEA:FSA1(7:3) has better oil tolerance ability, anti-adsorption ability and aging resistance ability. CEA:FSA1(7:3) was recommended as foam agent for natural gas foam flooding in JD Oilfield and the concentration was optimized as 0.5 %.

In the work, EOR technologies concerned in our work included further cross-linked polymer (C-P) flooding, surfactant-polymer (S-P) flooding, and polymer enhanced foam (PEF) flooding. It was necessary to optimize the best EOR technology for JD oilfield, and characterize the potentiality of polymer enhanced foam (PEF) flooding for the kind of post cross-polymer flooding reservoirs.

## Experimental

### Experimental equipments

Chemical flooding displacement experiment system produced by the American TEMECO was used in the experiments. The experimental equipments included a gas–liquid injection system, the temperature control and pressure sensor system, the core clamping system and the separation and measurement system of produced fluid. The gas–liquid injection system was used to provide the fluid conditions for the chemical flooding experiments. Four kinds of fluid including polymer, surfactant, oil and formation water can be simultaneously injected. Also, the gas can be injected at the designed speed. The fluid and gas can be injected to the core clamping system, which included two cores with different permeability. This kind of design can reflect the vertical heterogeneity of real reservoirs. The temperature control and pressure sensor system can make sure that the experiments were carried out in the designed condition. The separation and measurement system was used to automatically segregate and measure the fluid produced at the core outlet.

**Fig. 1:**
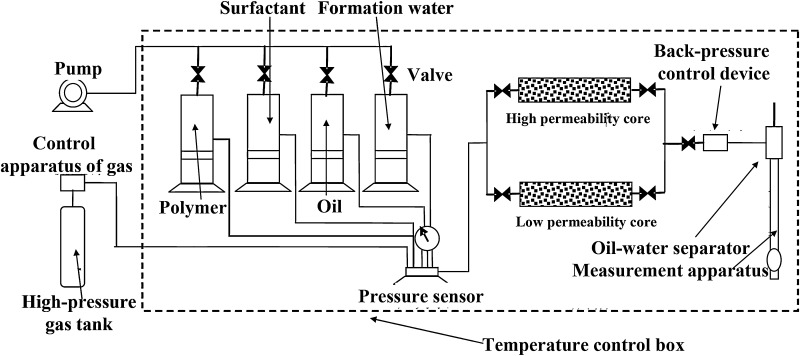
Experimental procedure for chemical flooding selection

The experimental procedure was shown in Fig. 1. The following ten items were included in the apparatus: (1) microscope constant-flux pump; (2) control apparatus of gas mass flow; (3) high-pressure gas tank; (4) storage tank of fluid agent; (5) pressure sensor; (6) two core models with different permeability; (7) back-pressure control device; (8) temperature control box; (9) separation apparatus; and (10) measurement apparatus.

In the chemical flooding selection experiments, the accuracy of the temperature was ±0.5 °C, the flow rate was limited to 0–30 mL in the control apparatus of gas mass flow, the pressure was limited to the range of 0–10 MPa in the back-pressure valve and the pressure-controlling accuracies of both the back-pressure valve and digital pressure gauge were 0.01 MPa.

### Materials

Materials used in experiments included natural gas, crude oil, AS-polymer, JRJL-3 crosslinker, JRJL-3 modifier, and surfactants. The natural gas and crude oil came from well G104-5 in JD reservoir. The crude oil had a viscosity of 95 mPa s and a density of 0.82 g/cm^3^. The component analysis result of natural gas was listed in Table 1.

**Table 1:** Component analysis result of natural gas

Components	Volume percentage/%	Components	Volume percentage/%
Methane	71.68	Isopentane	0.28
Ethane	11.47	*N*-pentane	0.27
Propane	6.67	Hexane	0.17
Iso-butane	0.86	Carbon dioxide	0.19
*N*-butane	1.38	Nitrogen	7.03

The JRJL-3 crosslinker, JRJL-3 modifier, AS-polymers were supported by JD oilfield. The JRJL-3 crosslinker, JRJL-3 modifier, AS-polymers were used in the actual cross-linked polymer (C-P) flooding process of JD reservoir. The surfactant system, CEA:FSA1(7:3), was screened in our former work. And it has good compatibility with natural gas. The physical properties of main chemical agents involved in the work were listed in Table 2.

**Table 2:** Physical properties of chemical agents

Cross-linked polymer	Molecular weight	Polymer content	Hydrolyzing degree	Intrinsic viscosity	Surfactant system	Effective content	PH valve	Density
	/	%	%	mL/g		%	/	kg/L
AS polymer	1.50 × 10^7^	92	25	1700	CEA:A1(7:3)	45	7–8	1.00–1.01

The parallel core system which contained cores with relatively high and low permeability. The parallel core system was a sand-packed tube model of 30 cm in the length and 2.5 cm in the diameter. The sand used in this work was siliceous sand, eighty percent of which in size was between 45 and 50 mesh. For each test, fresh sand was packed to ensure the same wettability. The following procedure was sand packing. And then measure the air permeability of the model. The absolute permeability of high permeability core was approximately 2500 × 10^−3^ and 600 × 10^−3^ μm^2^ in the low permeability core. Then saturate the model with brine water. Water-saturated process continued for 5 h, and the following oil-saturated process continued for more than 10 h. The oil saturation of models was absolute 80 % after oil-saturated process. The parallel core parameters are listed in Table 3.

**Table 3:** Parameters of parallel cores for experiments

Methods after cross-linked polymer flooding	(a) Further water flooding	(b) Cross-link polymer flooding	(c) Surfactant-polymer flooding	(d) Enhanced foam flooding
Model parameters				
Porosity/%	34.8	34.6	35.7	33.4
Permeability of high permeability core/10^−3^ μm^2^	2503	2494	2397	2544
Permeability of low permeability core/10^−3^ μm^2^	601	606	618	607
Oil saturation/%	82.1	84.4	86.3	78.8

### Experimental procedure

#### Scheme design

Four experimental schemes were designed in this study, as shown in Table 4. Each experiment had the first cross-linked polymer flooding process. After the first cross-linked polymer flooding process, EOR technologies concerned in our work included further water flooding, cross-linked polymer (C-P) flooding, surfactant-polymer (S-P) flooding, and polymer enhanced foam (PEF) flooding. Injection parameters of different chemical methods for each scheme were illustrated in Table 5.

**Table 4:** Experimental schemes

Number	Name	Experiments
a	/	Water flooding + first cross-linked polymer flooding + following water flooding
b	C-P	Water flooding + first cross-linked polymer flooding + following water flooding *+* *cross-linked polymer flooding* + following water flooding
c	S-P	Water flooding + first cross-linked polymer flooding + following water flooding + *surfactant-polymer (S-P) flooding* + following water flooding
d	PEF	Water flooding + first cross-linked polymer flooding + following water flooding + *polymer enhanced foam (PEF) flooding* + following water flooding

**Table 5:** Injection parameters of different chemical methods

Chemical flooding	Injection system	Pore volume
First cross-linked polymer flooding	AS polymer (2000 mg/L) + JRJL-3 crosslinker (800 mg/L) + JRJL-3 modifier (600 mg/L)	0.2
Further cross-linked polymer flooding	AS polymer (2000 mg/L) + JRJL-3 crosslinker (800 mg/L) + JRJL-3 modifier (600 mg/L)	0.3
Surfactant-polymer (S-P) flooding	AS polymer (2000 mg/L) + CEA:A1(7:3) (0.5 %)	0.3
Polymer enhanced foam (PEF) flooding	AS polymer (1500 mg/L) + surfactant [CEA:A1(7:3)] (0.5 %) + natural gas (gas liquid ratio 1:1)	0.3

As a comparative experiment, there was no chemical agent injected in the further water flooding experiment. The chemical agent used in the further cross-linked polymer (C-P) flooding was same with the actual cross-linked polymer in JD reservoir. The surfactant agent used in the further surfactant-polymer (S-P) flooding and polymer enhanced foam (PEF) flooding was CEA:FSA1(7:3), which was selected for natural gas in our former work. Foam performance evaluation experiments showed that CEA:FSA1(7:3) had good oil tolerance ability, anti-adsorption ability and aging resistance ability. These experiments were conducted at a temperature of 65 °C and the injection rate was set to 0.5 mL/min.

#### Experimental procedure

There were four procedures in the four experiments, including experimental apparatus connection, sand-packed model preparation, first cross-linked polymer flooding and further chemical flooding. Following are details.

1. Experimental apparatus connection

Connect the experimental apparatus according to Fig. 1.

2. Sand-packed model preparation

Saturate the sand-packed model with water, and flood the model with oil until the irreducible water saturation was reached.

3. First cross-linked polymer flooding
(1) Water flooding

Flood the model with water at a constant rate until the water cut of outlet reaches 94 %.

(2) First cross-linked polymer flooding

Inject cross-linked polymer system of 0.2 PV into the parallel core system at a constant rate.

(3) Following water flooding

Flood the model with water at a constant rate until the water cut of outlet reached 97 %.

4. Further chemical flooding
(1) Chemical slug injection

Inject the chemical slug of 0.3 PV into the model at a constant rate according to Table 5.

(2) Following water flooding

Flood the model with water at a constant rate until the water cut of outlet reached 98 %. End the experiment and prepare for the next one.

## Result analysis

### Water cut and recovery curves

**Fig. 2:**
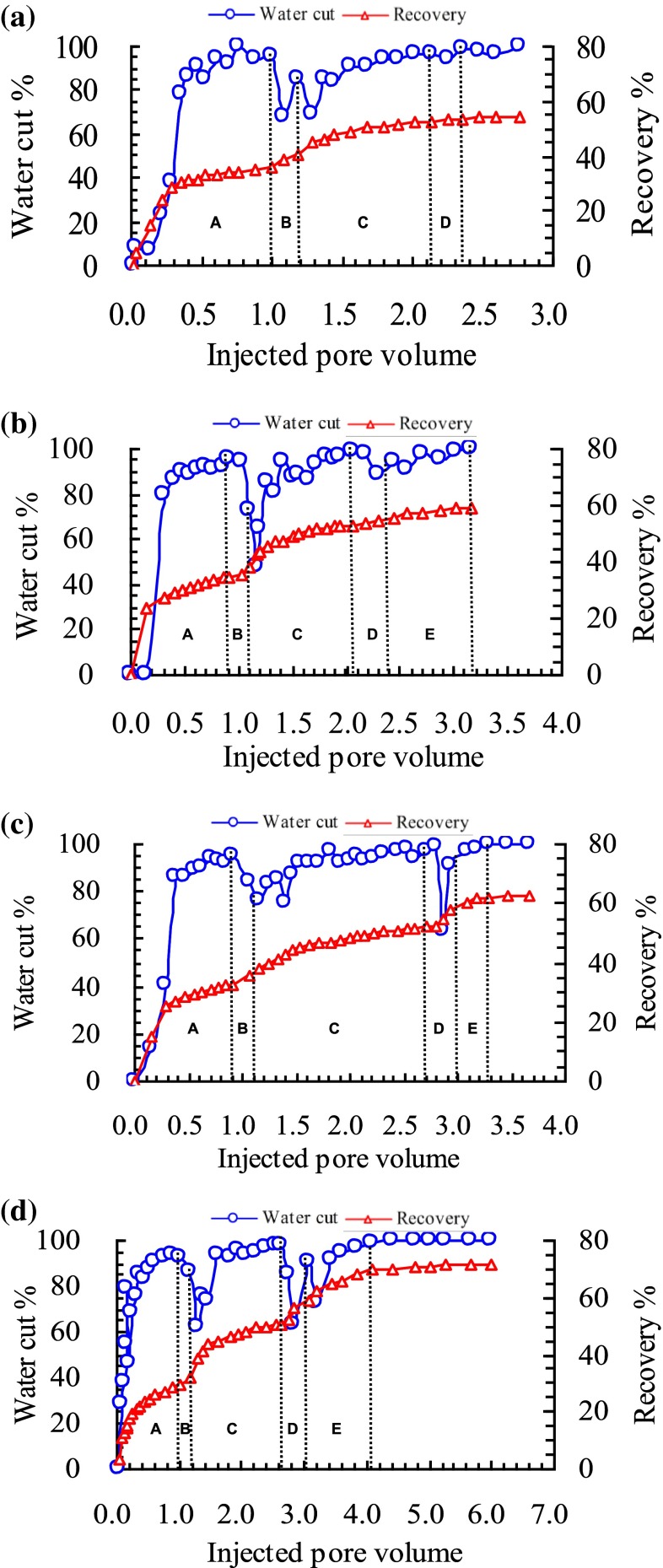
**a** Water cut and recovery curves of water flooding after cross-link polymer flooding. **b** Water cut and recovery curves of cross-link polymer flooding after cross-link polymer flooding. **c** Water cut and recovery curves of surfactant-polymer flooding after cross-link polymer flooding. **d** Water cut and recovery curves of enhanced foam flooding after cross-link polymer flooding. *A* Water flooding, *B* first cross-linked polymer flooding, *C* following water flooding until 97 % water cut, *D* following water flooding until 98 % water cut (**a**), further cross-linked polymer flooding (**b**), surfactant-polymer flooding (**c**), enhanced foam flooding (**d**), *E* following water flooding until 98 % water cut

The change curves of water cut and recovery in different chemical flooding experiments can reflect the development effect and potential EOR for the post cross-polymer reservoirs. The change curves of water cut and recovery in four different chemical flooding experiments was shown in Fig. 2a–d.

The cross-polymer flooding was carried out in all the four experiments when the water cut got 94 % during the water flooding process. It can be seen that the recovery of four different experiments, respectively, reached 35.8, 34.8, 32.6 and 29.3 % before the cross-polymer flooding was carried out. Then 0.2 PV of cross-polymer fluid was injected in every experiment followed by water flooding until the water cut reached 97 %. At the time, the recovery of four different experiments reached 53.2, 52.9, 52.1, 50.4 %, respectively. Each experiment has the roughly same amount of remaining oil before the cross-polymer flooding and after that. And the enhanced oil recovery of the four different experiments respectively reached 17.4, 18.1, 19.5, and 21.1 % during the cross-polymer flooding process.

**Fig. 3:**
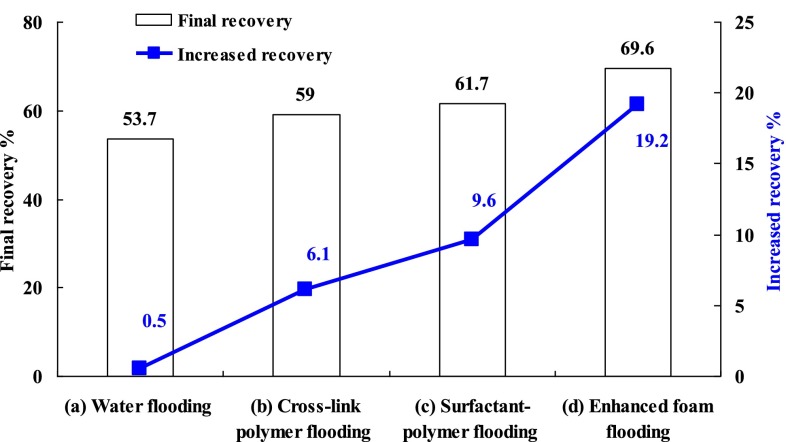
Final recovery and increased recovery for chemical methods after cross-link polymer flooding

It was different for the four experiments in the post cross-polymer flooding process. As a comparative experiment, there was no chemical agent injected in the experiment (a). And the water cut curve kept increasing until the end with the final recovery of 53.7 %. Cross-polymer solution slug of 0.3 PV was injected followed by water flooding until the water cut reached 98 % in the experiment (b). During the secondary cross-polymer flooding, the water cut curve decreased to 88.1 with 8.9 % down. Compared with the recovery before the secondary cross-polymer flooding, the final recovery at the end of experiment (b) reached 59.0 with 6.1 % increased. Surfactant-polymer solution slug of 0.3 PV was injected followed by water flooding until the water cut reached 98 % in the experiment (c). During the surfactant-polymer flooding, the water cut curve decreased to 63.4 with 33.6 % dropped. Compared with the recovery before surfactant-polymer flooding, the final recovery at the end of experiment (c) reached 61.7 with 9.6 % increased. Surfactant-polymer solution slug of 0.3 PV and natural gas with 1-1 gas liquid ratio were injected followed by water flooding until the water cut reached 98 % in the experiment (d). During the enhanced foam displacement stage, the water cut curve decreased 33.4 % with the most wide water drop funnel. Compared with the recovery before enhanced foam flooding, the final recovery at the end of experiment (d) reached 69.6 with 19.2 % increased. It can be concluded that the enhanced foam flooding had the best EOR effect for the post cross-polymer flooding reservoirs. The final recovery and increased recovery for chemical methods after cross-link polymer flooding was shown in Fig. 3.

### Liquid fraction comparison

**Fig. 4:**
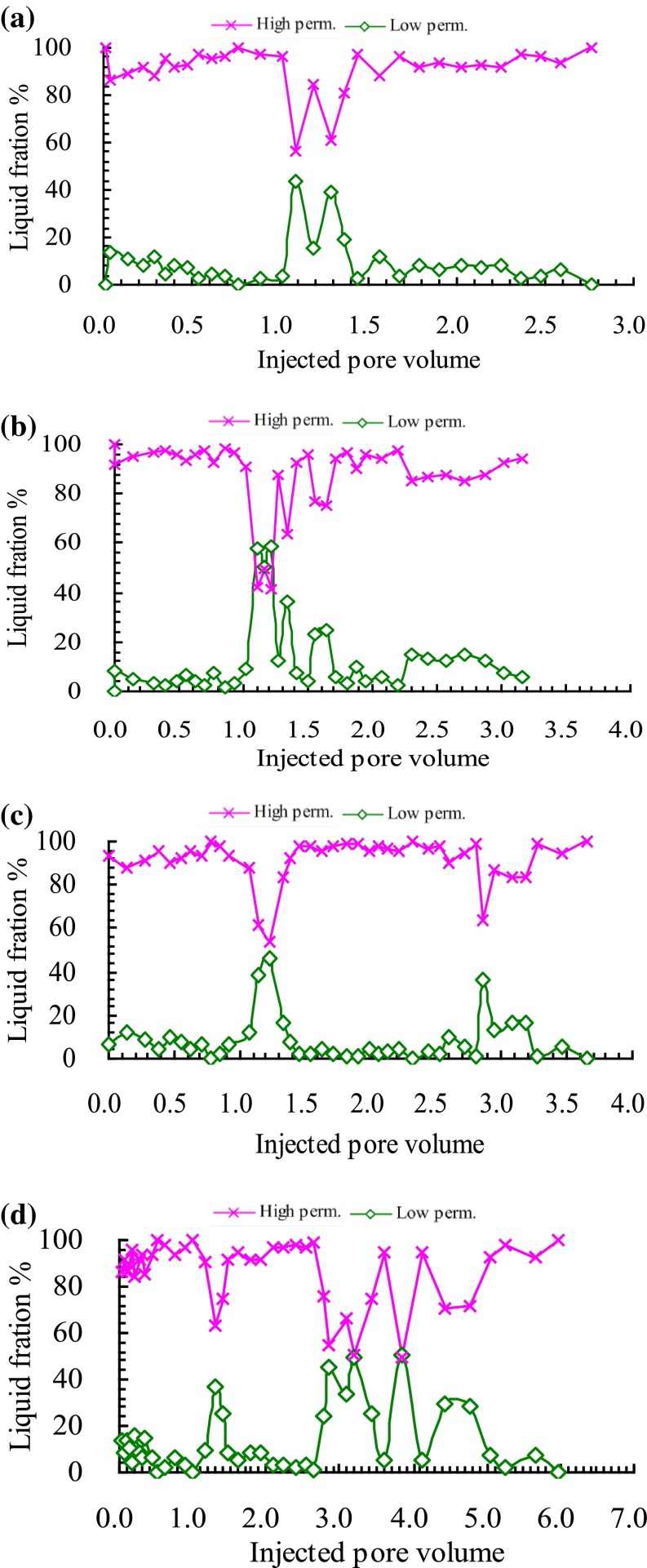
Liquid fraction curves of high and low permeability cores for **a** water flooding after cross-link polymer flooding, **b** cross-link polymer flooding after cross-link polymer flooding, **c** surfactant-polymer flooding after cross-link polymer flooding, **d** enhanced foam flooding after cross-link polymer flooding

Liquid fraction of cores means the flow rate ratio at the outlet of the high permeability and low permeability cores. It can characterize the chemical profile blocking effect. The liquid fraction comparison of high permeability core and low permeability core in the four experiments was shown in Fig. 4a–d.

**Fig. 5:**
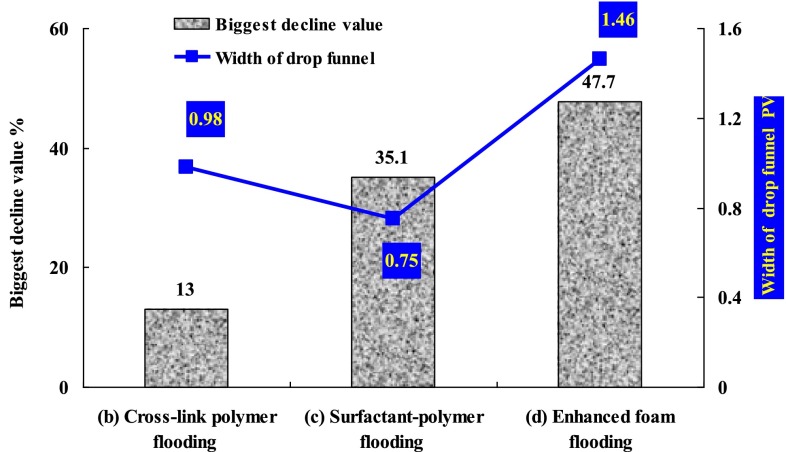
Biggest decline value and width of drop funnel for chemical methods after cross-link polymer flooding

From the initial stage of four experiments, it can be seen that the liquid fraction of high permeability core remain at more than 90 %, while less than 10 % for the low permeability core. Compared with the low permeability core, the injected water preferred to flow in the high permeability core at the same pressure difference. With the thief zone formed in the high permeability, there was more and more fluid flowing in the high permeability core, and there was less and less injected fluid flowing in the low permeability. The water cut of experiments got higher and higher. Then cross-polymer fluid of 0.2 PV was injected in every experiment followed by water flooding until the water cut reached 97 %. The cross-polymer solution preferred to flow in the thief zone of high permeability core, and played plugging effect. It can be seen that the liquid fraction of the high permeability core decreased, while the liquid fraction of low permeability increased during the cross-polymer flooding process in the four experiments. During the following water flooding stage after the first cross-polymer flooding, the new thief zone was formed and the liquid fraction of high permeability core increased to more than 90 %. Meanwhile, there was fluid of less than 10 % flowing through the low permeability core. In the post cross-polymer flooding process, liquid fraction curves of high and low permeability cores for the four experiments were different because different chemical fluid was injected in the four experiments. As a comparative experiment, there was no chemical agent injected in the experiment (a). And the liquid fraction of high permeability core kept increasing until the end with the final fraction of 100 %. The change trend of liquid fraction in the low permeability was opposite with that in the high permeability. In the post cross-polymer flooding process, further cross-polymer solution slug of 0.3 PV was injected in the experiment (b). As shown in Fig. 4b, a drop funnel of liquid fraction in high permeability core was formed during the further cross-linked polymer flooding stage. The liquid fraction of high permeability core decreased from 97.8 to 84.8 % with the biggest decline value of 13.0 %. And then the liquid fraction of high permeability core increased slowly along with the PV number of following water flooding increased. In the further cross-linked polymer flooding process, the width of the funnel was 0.98 PV. The biggest decline value and the width of the drop funnel reflect the selective plugging effect of different chemical methods. In the experiment (c), surfactant-polymer solution slug of 0.3 PV was injected. In the experiment (d), surfactant-polymer solution slug of 0.3 PV and natural gas with 1-1 gas liquid ratio were injected. The biggest decline value and the width of the drop funnel of experiment (b–d) were shown in Fig. 5. It can be seen that there was the biggest decrement (47.7 %) in experiment (d), also the decrement last the longest PV (1.46 PV). It indicated that the enhanced foam flooding had the best selective blocking effect for the post cross-polymer flooding reservoirs.

### Recovery comparison

**Fig. 6:**
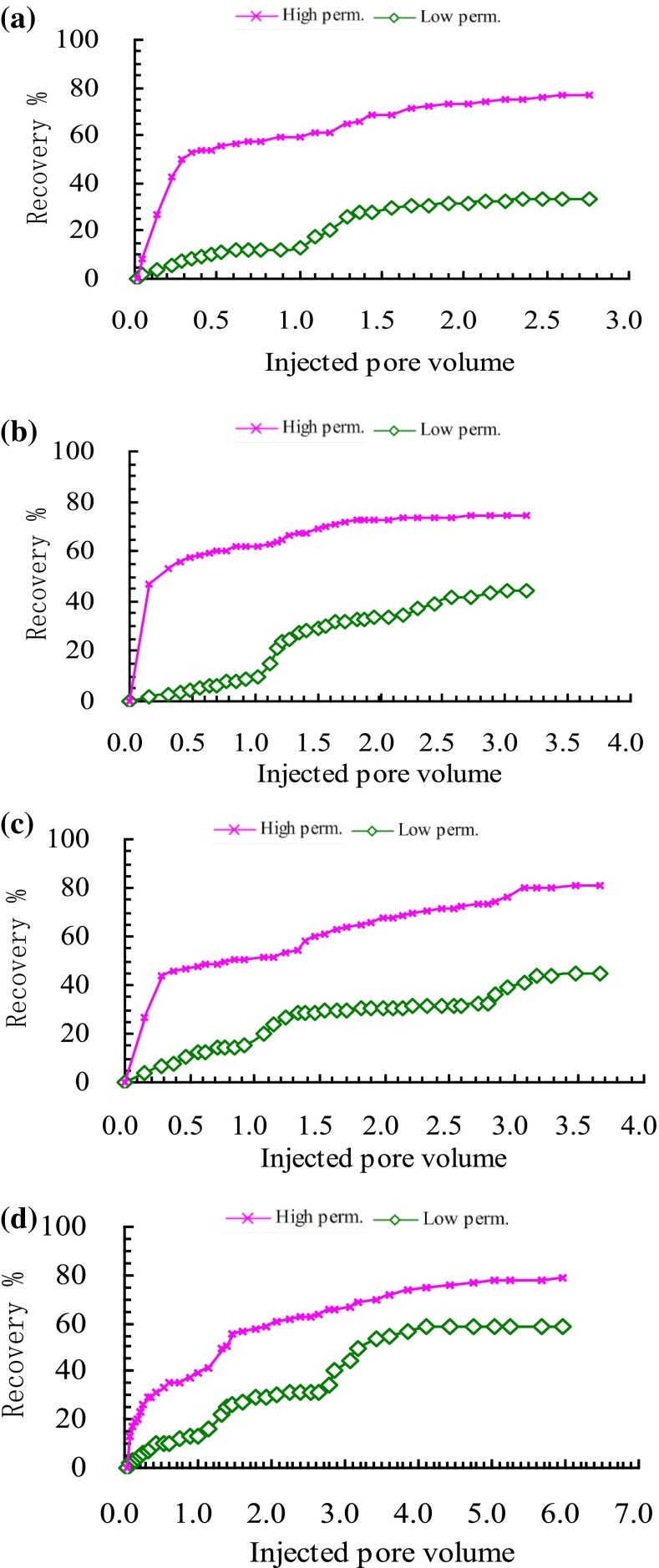
Recovery curves of high permeability and low permeability cores for **a** water flooding after cross-link polymer flooding, **b** cross-linked polymer flooding after cross-link polymer flooding, **c** surfactant-polymer flooding after cross-link polymer flooding, **d** enhanced foam flooding after cross-link polymer flooding

Liquid fraction of cores can characterize the chemical profile blocking effect. However, it cannot reflect the displacement oil ability of chemical agent in different experiments for the post-polymer flooding reservoirs. The recovery comparison of high permeability core and low permeability core in the four experiments was shown in Fig. 6a–d.

**Fig. 7:**
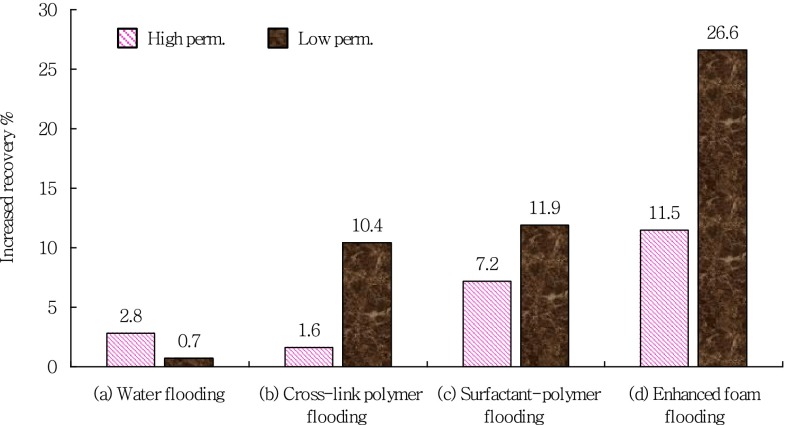
Increased recovery of high permeability and low permeability cores for chemical methods after cross-link polymer flooding

It can be found that the recovery of high permeability core was obviously higher than low permeability core in each experiment. From experiment (a) to experiment (b), the recovery of high permeability core were 76.5, 74.4, 80.1, 74.6 %. Figure 7 gave the increased recovery of high permeability and low permeability cores for chemical methods after cross-link polymer flooding. Compared with the cross-polymer flooding process, the increased recovery of high permeability core were 2.8, 1.6, 7.2, 11.5 %, respectively. The enhanced oil recovery of experiment (c) and experiment (d) were higher, which indicated that CEA:A1(7:3) system screened had a good ability to displace oil because of its good ability to reduce the interfacial tension. From experiment (a–b), the recovery of low permeability core was 33.2, 44.1, 44.0, 58.4 %. Compared with the cross-polymer flooding process, the increased recovery of low permeability core were 0.7, 10.4, 11.9, 26.6 %, respectively. The enhanced oil in the further cross-polymer flooding experiment mainly come from the low permeability core. Compared with the cross-polymer flooding experiment, there was no big difference in the recovery of low permeability core of the surfactant-polymer flooding experiment. It indicated that the surfactant injected did not play good washing oil effect in the low permeability core. Surfactant-polymer solution slug of 0.3 PV and natural gas with 1-1 gas liquid ratio were injected followed by water flooding until the water cut reached 98 % in the experiment (d). Foam with a high apparent viscosity formed in the high and low permeability cores during the injection process. And it played the role of selectively blocking in the high permeability core, and displaced the oil in the low permeability core at the same time. Also, as surfactant with good ability of reducing interfacial tension in the enhanced foam system, CEA:A1(7:3) can increase capillary number, accordingly, reduce saturation of irreducible oil and enhance oil recovery in the high and low permeability cores. Based on the above analysis, enhanced foam system can simultaneously enlarge sweep volume and increase washing efficiency in the high and low permeability cores. Considering selective plugging feature and good washing oil ability, polymer enhanced foam (PEF) flooding was taken as following EOR technology for JD reservoir.

## Conclusion

1. To compare the recovery efficiency by different EOR technologies, series of oil displacement experiments were carried out in a parallel core system which contained cores with relatively high and low permeability. EOR technologies concerned in our work include further cross-linked polymer (C-P) flooding, surfactant-polymer (S-P) flooding, and polymer enhanced foam (PEF) flooding. Results showed that PEF flooding had the highest enhanced oil recovery of 19.2 % original oil in place (OOIP), followed by S-P flooding (9.6 % OOIP) and C-P flooding (6.1 % OOIP).
2. Produced liquid percentage results indicated PEF flooding can more efficiently promote the oil recovery in the lower permeability core by modifying the injection profile.
3. Considering selective plugging feature and good washing oil ability, polymer enhanced foam (PEF) flooding was taken as following EOR technology for JD reservoir.
